# Morroniside induces cardiomyocyte cell cycle activity and promotes cardiac repair after myocardial infarction in adult rats

**DOI:** 10.3389/fphar.2023.1260674

**Published:** 2024-01-11

**Authors:** Songyang Zheng, Tingting Liu, Mengqi Chen, Fangling Sun, Yihuan Fei, Yanxi Chen, Xin Tian, Zheng Wu, Zixin Zhu, Wenrong Zheng, Yufeng Wang, Wen Wang

**Affiliations:** ^1^ Department of Experimental Animal Laboratory, Xuanwu Hospital of Capital Medical University, Beijing, China; ^2^ School of Chemical and Pharmaceutical Engineering, Hebei University of Science and Technology, Shijiazhuang, Hebei, China; ^3^ Department of Functional Neurosurgery, Xuanwu Hospital Capital Medical University, Beijing, China; ^4^ Beijing Institute for Brain Disorders, Beijing, China

**Keywords:** cardiac repair, cell cycle, morroniside, myocardial infarction, myocardial regeneration

## Abstract

**Introduction:** Acute myocardial infarction (AMI) is characterized by the loss of cardiomyocytes, which impairs cardiac function and eventually leads to heart failure. The induction of cardiomyocyte cell cycle activity provides a new treatment strategy for the repair of heart damage. Our previous study demonstrated that morroniside exerts cardioprotective effects. This study investigated the effects and underlying mechanisms of action of morroniside on cardiomyocyte cell cycle activity and cardiac repair following AMI.

**Methods:** Neonatal rat cardiomyocytes (NRCMs) were isolated and exposed to oxygen-glucose deprivation (OGD) *in vitro*. A rat model of AMI was established by ligation of the left anterior descending coronary artery (LAD) *in vivo*. Immunofluorescence staining was performed to detect newly generated cardiomyocytes. Western blotting was performed to assess the expression of cell cycle-related proteins. Electrocardiography (ECG) was used to examine pathological Q waves. Masson’s trichrome and wheat germ agglutinin (WGA) staining assessed myocardial fibrosis and hypertrophy.

**Results:** The results showed that morroniside induced cardiomyocyte cell cycle activity and increased the levels of cell cycle proteins, including cyclin D1, CDK4, cyclin A2, and cyclin B1, both *in vitro* and *in vivo*. Moreover, morroniside reduced myocardial fibrosis and remodeling.

**Discussion:** In conclusion, our study demonstrated that morroniside stimulates cardiomyocyte cell cycle activity and cardiac repair in adult rats, and that these effects may be related to the upregulation of cell cycle proteins.

## 1 Introduction

Acute myocardial infarction (AMI) is considered a leading cause of morbidity and mortality worldwide (Mensah et al., 2019). Patients with AMI exhibit cardiomyocyte loss or dysfunction caused by ischemia and hypoxia (Nakada et al., 2017; Hesse et al., 2018). Dead myocardial tissue is digested by immune cells and replaced by fibrosis, leading to myocardial remodeling, heart failure, arrhythmia, and death (Prabhu and Frangogiannis, 2016). Despite notable breakthroughs in numerous therapeutic strategies, including percutaneous catheter intervention and surgical bypass grafting (Samsky et al., 2021; Shi et al., 2022), the present treatments only temporarily enhance heart function and do not replace lost cardiomyocytes.

In non-mammalian vertebrates, including zebrafish, newts, and axolotls, the regenerative capacity of cardiomyocytes persists throughout life (Hu et al., 2022). In contrast to lower vertebrates, adult mammalian cardiomyocytes lose their mitotic capacity following a narrow proliferation window in the neonatal stages (Porrello et al., 2011) and have limited myocardial regeneration capacity after injury; the renewal rate of these cardiomyocytes is less than 1% per year (Mahmoud et al., 2014a; Gao et al., 2019). Even though myocardial regeneration is enhanced after AMI, it cannot compensate for the massive loss of cardiomyocytes or restore myocardial function in injured hearts. As a result, the detection of novel drugs or molecular mechanisms that promote cardiomyocyte proliferation after AMI is urgently needed. Regulation of cell cycle activation and re-entry can induce cardiomyocyte division and proliferation. Bae et al. showed that malonate promoted adult cardiomyocyte cell cycle re-entry and proliferation and heart regeneration through metabolic reprogramming (Jiyoung et al., 2021). Wu et al. showed that low-density lipoprotein receptor-related protein 6 (LRP6) regulates cardiomyocyte proliferation via the ING5/P21 signaling pathway, and that elevated cardiomyocyte proliferation mediated by LRP6 deficiency results in adult myocardial repair and improvement in heart function following AMI (Wu et al., 2021). These studies show that stimulating cell cycle activation of terminally differentiated cardiomyocytes is a feasible approach for treating AMI.

Morroniside, which is extracted from *Cornus Officinalis*, is one of the most abundant iridoid glycosides. Previous studies have shown that morroniside attenuates inflammation, protects cardiomyocytes, improves cardiac function following AMI, and promotes rat coronary artery endothelial cell proliferation (Yu et al., 2018; Liu et al., 2020; Liu et al., 2022). Nevertheless, there is no information available regarding the potential function of morroniside in cardiomyocyte cell cycle activity and repair. Based on a rat model of AMI, the current study was designed to study the effects of morroniside on cardiomyocyte cell cycle activity and to further investigate the underlying mechanisms. Moreover, our results provide an experimental foundation to further explain the function of morroniside in cardiomyocyte cell cycle activity after AMI.

## 2 Methods

### 2.1 Preparation of drug

In this study, we extracted purified morroniside from *C. Officinalis* sarcocarp provided by Tong-Ren-Tang (Beijing, China), in accordance with a previously described method (Wang et al., 2010). High-performance liquid chromatography (HPLC) was used to obtain a final purity of 98.5%.

### 2.2 Animals

Animal experiments were approved by the Animal Care and Welfare Committee of Xuanwu Hospital, Capital Medical University, China (approval number XW-20210501-01). All surgeries were performed under anesthesia, and animal suffering was minimized as much as possible. Male Sprague-Dawley rats, aged 6–8 weeks and weighing 260-280 g, were provided by the Beijing Vital River Experimental Animal Co. (Beijing, China) and raised at 22°C ± 2°C with a 12-h/12-h light/dark cycle. The rats were provided free access to food and water.

### 2.3 Neonatal rat primary cardiomyocytes (NRCMs) isolation and culture

NRCMs were obtained from newborn rats (1–2 days after birth). Briefly, heart tissues were collected from newborn rats and washed with D-Hank’s solution. Subsequently, the heart tissues were minced prior to digestion with trypsin (0.075%; Gibco, Thermo Fisher Scientific Inc., Waltham, MA, United States) and type II collagenase (0.1%; Solarbio, Beijing, China). To suspend the cells, high-glucose Dulbecco’s modified Eagle’s medium (DMEM) (Gibco, Thermo Fisher Scientific) containing 100 U/mL of penicillin, 100 mg/mL streptomycin (Gibco, Thermo Fisher Scientific), and 10% fetal bovine serum (FBS, Biological Industries, Kibbutz Beit-Haemek, Israel) was added. Then, cells were seeded on 10-cm plastic dishes in a humid incubator for a 1.5-h period under 5% CO_2_ and 37°C conditions. DMEM-suspended cells were inoculated in glass-bottom cell culture dishes or 48-well plates at 20,000 cells/well in 5% CO_2_ in a humidified atmosphere at 37°C.

### 2.4 Oxygen-glucose deprivation (OGD)

To simulate ischemia *in vitro*, we exposed cells to hypoxia (95% N_2_ together with 5% CO_2_) prior to incubation for 6 h in glucose-free DMEM (Gibco, Thermo Fisher Scientific), and exposed cells in the control group to normoxia (95% air and 5% CO_2_) for 6-h incubation. The cells were classified into six groups. We then cultured control cells normally and treated OGD cells with OGD for a 6-h period. Cells in the control + morroniside group were pretreated with 100 μM morroniside for 24 h. Cells in the three OGD + morroniside groups were subjected to 24-h pretreatment with 1, 10, or 100 μM morroniside prior to 6-h of OGD. After cell harvesting, radioimmunoprecipitation assay (RIPA) lysis buffer (Applygen, Beijing, China) containing phosphatase inhibitors (Roche, Indianapolis, IN, United States) was added to lyse the cells for Western blot analysis. For immunofluorescence staining analysis, the cells were fixed with 4% paraformaldehyde (PFA) for 20 min before immunofluorescence analysis.

### 2.5 AMI model establishment

As previously described (Liu et al., 2020), the rats underwent tracheal intubation, followed by ventilation with 3% isoflurane for induction and 2% isoflurane for anesthesia maintenance. Thoracotomy was performed in the fourth left intercostal space to expose the heart. We permanently ligated the left anterior descending (LAD) coronary artery 2 mm beneath the left atrium using a 6-0 prolene suture and closed the chest postoperatively. The rats were warmed for 15 min until they recovered. Rapid anterior surface discoloration and ST-segment elevation on electrocardiography (ECG) indicated a successful operation. Rats in the sham operation, AMI, and morroniside-treated AMI groups were administered morroniside at 60, 120, or 240 mg/kg. Specifically, morroniside was dissolved in normal saline before intragastric administration once daily from 3-h post-AMI for 7 and 28 days. Equivalent volumes of normal saline were administered to the sham-operation and AMI groups. The hearts were dissected 7 and 28 days after the operation for further analysis.

### 2.6 5-bromo-2-deoxyuridine (BrdU) incorporation assay

NRCMs were cultured in a medium containing 10 μM BrdU (Sigma-Aldrich, St. Louis, MO, United States) a day before OGD or normal culture, followed by another 6-h incubation with BrdU. In the adult heart section BrdU assay, BrdU at 500 μg/mL (Sigma-Aldrich) was administered to each rat via intraperitoneal injection twice a day after surgery for a 7-day period.

### 2.7 Immunofluorescence staining

After 15-min of fixation using 4% PFA, cultured cells were rinsed with 0.1 M phosphate-buffered saline (PBS) three times for 5 min each. Later, 20-min permeabilization was performed with 0.1% Triton X-100 at ambient temperature. Subsequently, 5% donkey serum (Jackson ImmunoResearch Laboratories) was added to block the cells at ambient temperature for 1 h, followed by overnight incubation with primary antibodies at 4°C. After washing with PBS, the cells were incubated for 2 h with Alexa Fluor-488- (1:400, A21206, Life Technologies, Carlsbad, CA, United States) or Alexa Fluor-594-conjugated (1:400, A21203, Life Technologies) secondary antibodies at ambient temperature. After washing thrice with PBS, diamidino-2-phenylinidole (DAPI; Abcam, Cambridge, MA, United States) was added for nuclear staining. For BrdU staining, cells were permeabilized with 2 N HCl for 17 min at 37°C prior to blocking with donkey serum.

For heart slides, rats were killed at 7 and 28 days post-AMI, and the obtained myocardial samples were subjected to optimal cutting temperature (OCT) compound (Sigma-Aldrich) embedding and rapid freezing within liquid nitrogen prior to preservation at −80°C; 6-μm cryostat sections were cut. Ice-cold sections and neonatal rat primary cardiomyocytes were permeabilized with 0.1% Triton X-100 in PBS for 30 min, followed by 2 h of blocking using 5% donkey serum (Jackson ImmunoResearch Laboratories) at ambient temperature, as well as overnight primary antibody incubation at 4°C followed by incubation with Alexa Fluor-488 (1:400; Life Technologies) or Alexa Fluor-594-conjugated (1:400; Life Technologies) secondary antibodies. In addition, the nuclei were stained with mounting medium containing DAPI (Abcam). A Nikon 80i fluorescence microscope and Leika MICA confocal microscopy was used to visualize fluorescence signals. New positively stained cardiomyocytes per unit area were quantified using the Image-Pro Plus software package (version 6.0; IPP, Media Cybernetics, Maryland, United States).

The primary antibodies used in this assay were mouse anti-cTnT (1:200, ab8295, Abcam), rabbit anti-cTnT (1:200, ab209813, Abcam), rabbit anti-Ki67 (1:200, ab15580, Abcam), mouse anti-BrdU (1:200, 11170376001, Roche, Indianapolis, IN, United States), rabbit anti-histone H3 (1:200, ab5176, Abcam), rabbit anti-Aurora B (1:200, ab2254, Abcam) and anti-α-actinin (1:200, ab9465, Abcam).

### 2.8 Western blotting assay

NRCMs and hearts were homogenized and lysed in pre-chilled RIPA buffer (Applygen) containing phosphatase inhibitors (Roche) to extract proteins. The protein content was analyzed using a bicinchoninic acid protein assay kit (Applygen). Subsequently, equivalent amounts of total proteins (50 μg cardiac proteins and 70 μg cellular proteins) were blended prior to dissolution in 5 × sodium dodecyl-sulfate–polyacrylamide gel electrophoresis (SDS-PAGE) sample buffer and then heated to 95°C for 5 min. Following SDS-PAGE for protein separation, the separated proteins were transferred onto nitrocellulose membranes. Primary antibodies against cyclin D1 (1:1,000, ab134175; Abcam), cyclin-dependent kinase 4 (CDK4) (1:1,000, 09173-4F11; Sigma-Aldrich), cyclin A2 (1:1,000, ab181591; Abcam), and cyclin B1 (1:1,000, 4135S; Cell Signaling Technology, Beverly, MA, United States) were used for immunodetection. Subsequently, horseradish peroxidase-conjugated anti-rabbit or anti-mouse secondary antibodies were added. Finally, an ECL kit (Millipore, Temecula, CA, United States) was used to visualize immunoreactive proteins on the membrane.

### 2.9 Electrocardiogram

After ventilation with 3% and 2% isoflurane for anesthesia induction and maintenance, respectively, the rats were injected with needle electrodes in the lead II position. ECGs were recorded for each experimental mouse (MP150; BIOPAC Co., United States).

### 2.10 Wheat germ agglutinin (WGA)

After dissection, heart tissue was immersed in liquid nitrogen for snap-freezing using an OCT compound, cut into 6-μm sections, and fixed with acetone. After washing with PBS, the sections were blocked with 5% donkey serum before incubation with Alexa-488-conjugated WGA (1:500, W11261, Thermo Fisher Scientific). ImageJ software was used to measure cell size.

### 2.11 Masson’s trichrome staining

After collection, the heart tissue was subjected to 4% PFA fixation, embedded in OCT, and sliced into 6-μm sections. Subsequently, four sections were prepared on one slide. Masson’s trichrome staining was carried out in line with specific instructions. Serial sections from the apex to the ligation site were examined. The average percent fibrotic area of the total area was calculated using ImageJ software based on Masson’s trichrome staining to quantify scar size.

### 2.12 Statistical analysis

The results are presented as the mean ± standard error of the mean (SEM). Statistical analyses were conducted using SPSS 23.0 and Prism 5.0 (GraphPad Software). One-way ANOVA and Tukey’s test were used to compare multiple groups. The significance levels are indicated at *p* < 0.05, *p* < 0.01, and *p* < 0.001.

## 3 Results

### 3.1 Morroniside facilitates OGD NRCM cell cycle activity *in vitro*

**FIGURE 1:**
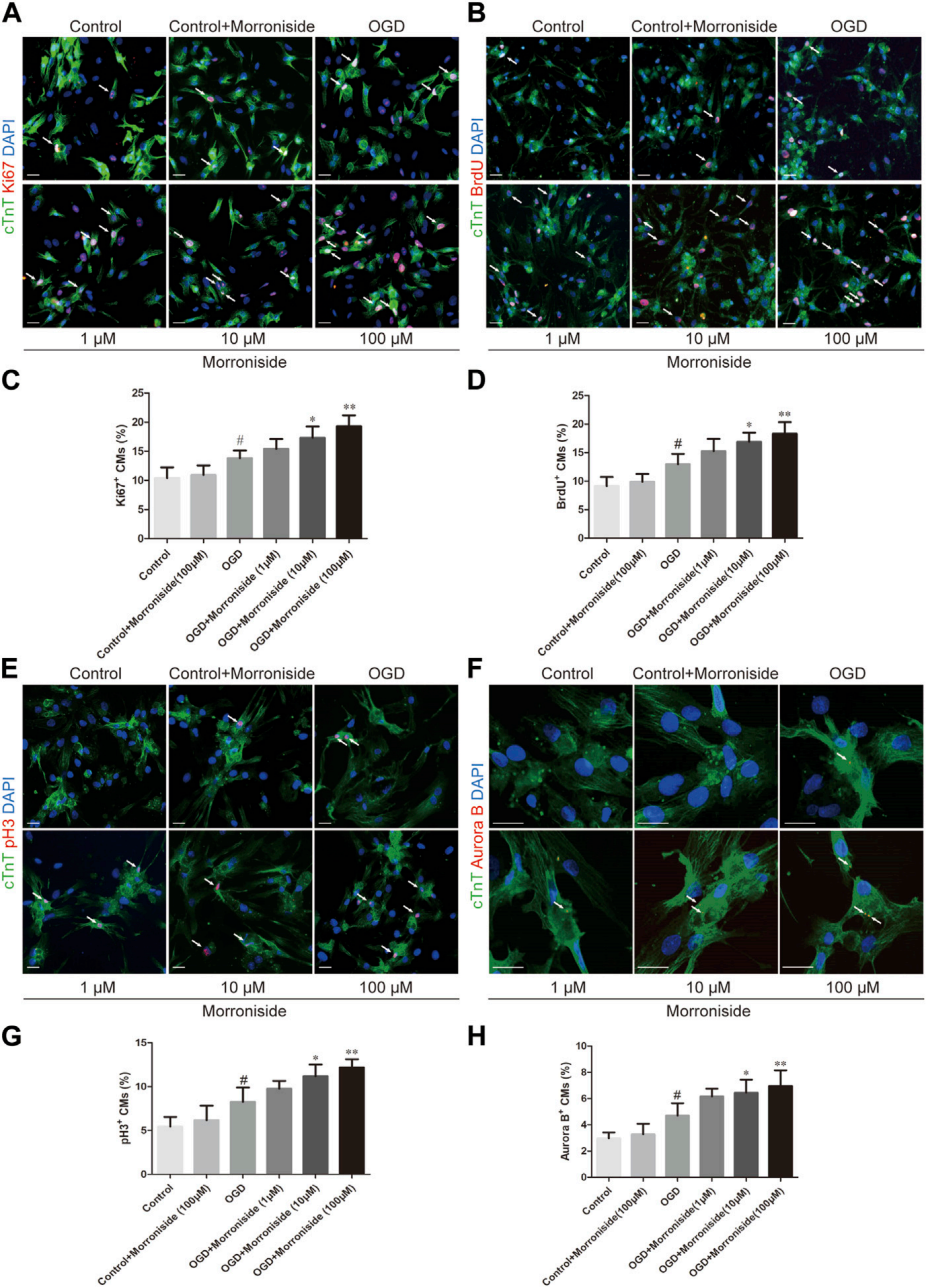
Morroniside induces the cell cycle activity of neonatal cardiomyocytes *in vitro*. **(A,B,E,F)** Representative images of NRCM cultures stained with Ki67/cTnT, BrdU/cTnT, pH3/cTnT, Aurora B/cTnT, respectively. **(C,D,G,H)** The column presents the ratio of cell cycle activated NRCMs (*n* = 5). Scale bar = 20 μm. Data are presented as the mean ± SEM. ^#^ *p* < 0.05, ^##^ *p* < 0.01, ^###^ *p* < 0.001 VS the control group; *p < 0.05, **p < 0.01, ***p < 0.001 relative to the OGD group.

To investigate whether morroniside induces cardiomyocyte cell cycle activity, we first labeled cell cycle activated NRCMs with Ki67 and cTnT using immunofluorescence staining. The number of cell cycle activated cardiomyocytes was notably higher in the OGD group than that in the control group. In addition, the number of Ki67^+^/cTnT^+^ cells was higher in the 10 and 100 μM morroniside-treated groups than in the OGD group (Figures 1A, C). We labeled the S phase with BrdU to further demonstrate that morroniside induced cardiomyocyte cell cycle activity (Yu et al., 2022). The 10 and 100 μM morroniside-treated groups exhibited a pronounced increase in the ratio of BrdU^+^/cTnT^+^ cells relative to the OGD group (Figures 1B, D). To determine whether increased DNA replication in cardiomyocytes ultimately leads to increased karyokinesis and cytokinesis, we stained the cells with phospho-histone H3 (pH3), a marker of late G2-phase/mitosis (Bhaskar et al., 2018), and Aurora B, a kinase localized in the midbody during cytokinesis (Gang et al., 2017). As indicated in Figures 1E–H, the percentages of pH3^+^/cTnT^+^ and Aurora B^+^/cTnT^+^ cells in the 10 μM and 100 μM morroniside-treated groups were higher than those in the OGD group. These results suggested that morroniside induced NRCM cell cycle activity after OGD *in vitro*.

### 3.2 Morroniside induces cardiomyocyte cell cycle activity in adult rat hearts 7 days after AMI

**FIGURE 2:**
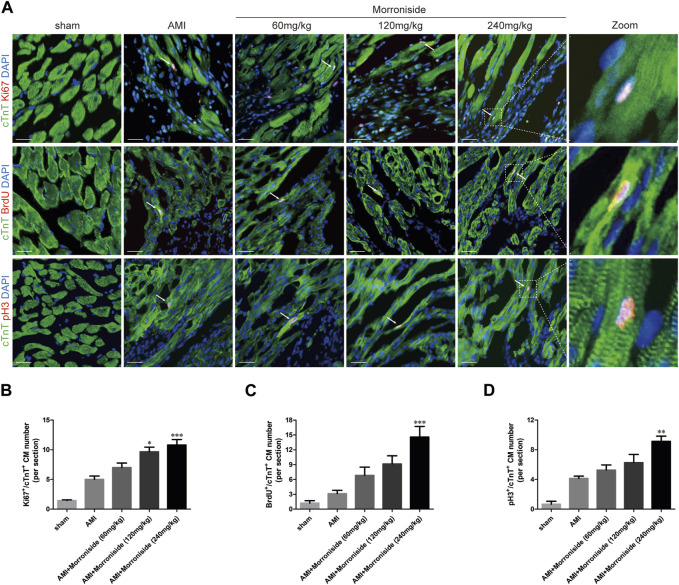
Morroniside induces cardiomyocyte cell cycle activity in adult rat hearts 7 days after AMI. **(A)** Representative immunostaining images of cell cycle activated cardiomyocytes which are labeled with Ki67, BrdU, pH3 and cTnT in the peri-infarcted myocardium a week following AMI. **(B–D)** Quantitative analysis of the number of Ki67^+^/cTnT^+^, BrdU^+^/cTnT^+^and pH3^+^/cTnT^+^ cell numbers (*n* = 3 rats in sham group, *n* = 5 rats in other groups). Scale bar = 20 μm. Data are presented as the mean ± SEM. *p < 0.05, **p < 0.01, ***p < 0.001 VS the AMI group.

**FIGURE 3:**
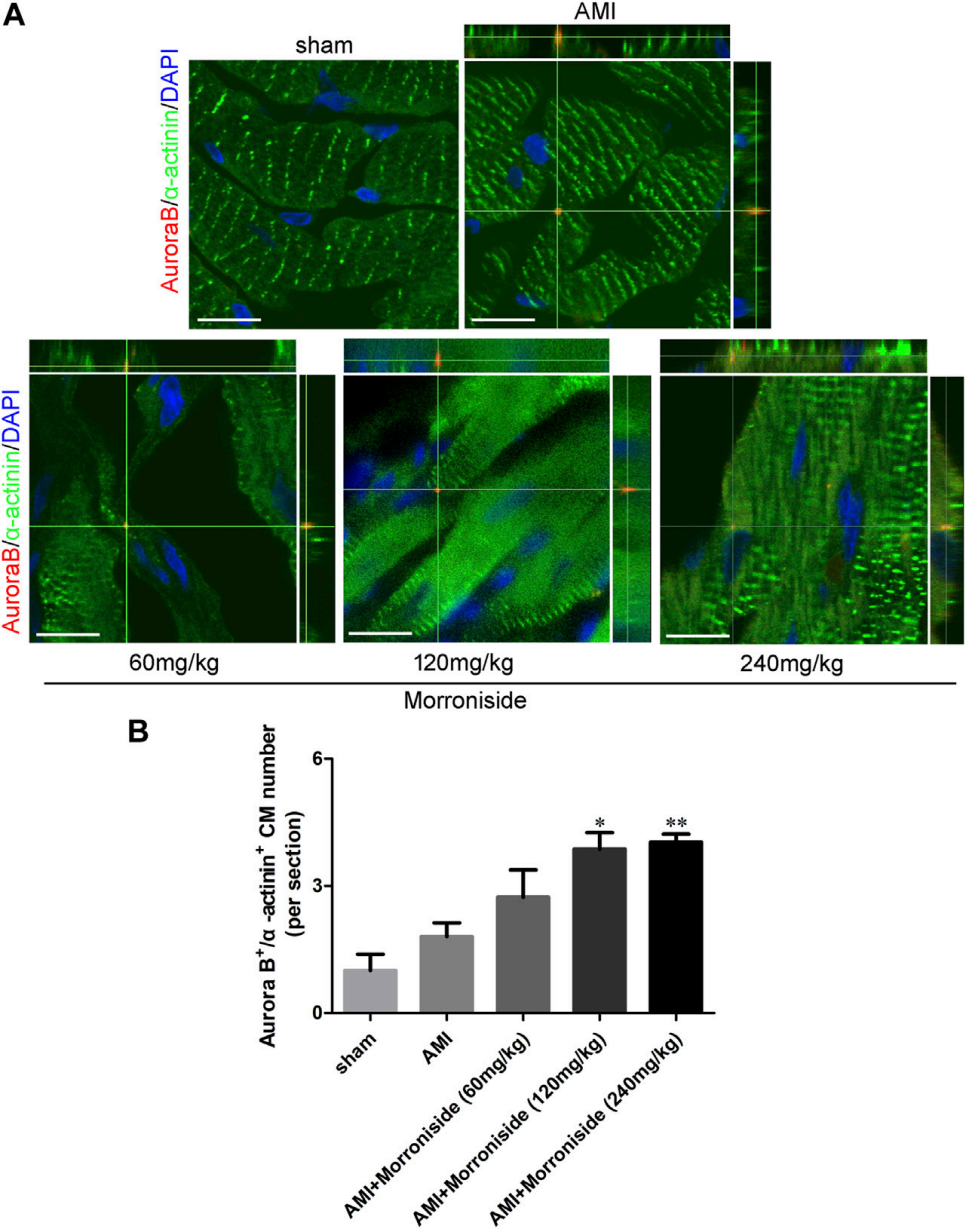
Morroniside promotes cardiomyocyte mitosis in adult rat hearts 7 days after AMI. **(A)** Representative immunostaining images of mitotic cardiomyocytes labeled Aurora B and α-actinin in the peri-infarcted myocardium a week following AMI. **(B)** Quantitative analysis of Aurora B^+^/α^−^actinin^+^ cell numbers (*n* = 3 rats in sham group, *n* = 5 rats in other groups). Scale bar = 15 μm. Data are presented as the mean ± SEM. *p < 0.05, **p < 0.01 VS the AMI group.

We further determined whether morroniside induces cardiomyocyte cell cycle activity after AMI *in vivo*. We evaluated Ki67, BrdU, pH3, and Aurora B expression in cTnT^+^ cardiomyocytes *in vivo* (Gao et al., 2019; Magadum et al., 2020; Liu et al., 2021; Wu et al., 2021; Li et al., 2023). As shown in Figures 2, 3, the number of cell cycle activated cardiomyocytes increased at the infarct border in the AMI group. Compared to the AMI group, morroniside treatment at doses of 120 and 240 mg/kg notably elevated Ki67^+^ (Figure 2B) and Aurora B^+^ (Figure 3B) cardiomyocytes, whereas morroniside treatment at 240 mg/kg notably elevated the number of BrdU^+^ (Figure 2C) and pH3^+^ cardiomyocytes (Figure 2D). These results suggest that morroniside induces cardiomyocyte cell cycle activity in adult rat hearts 7 days after AMI *in vivo*.

### 3.3 Morroniside stimulates the expression of proteins in association with the cell cycle *in vitro* and *in vivo*

**FIGURE 4:**
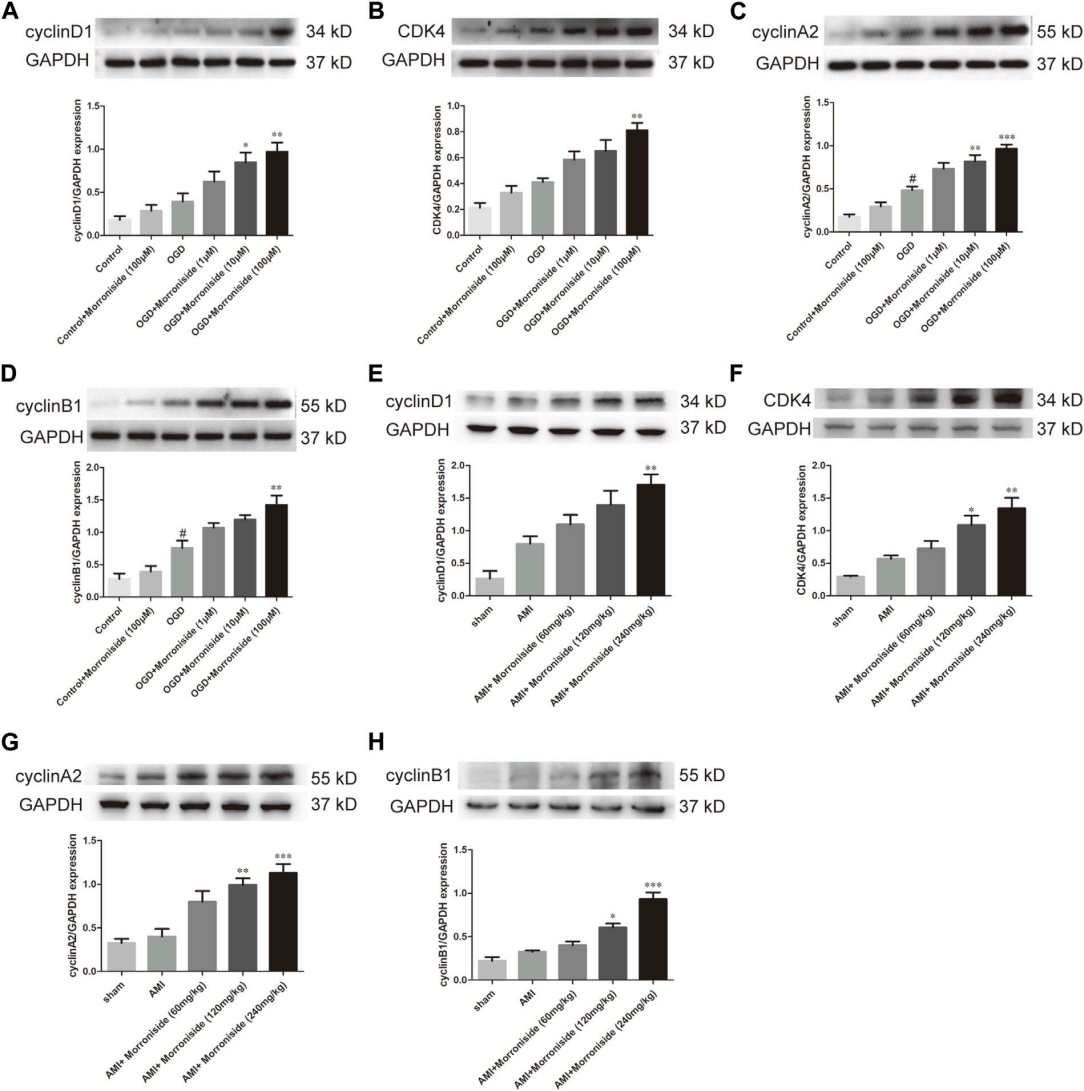
Morroniside stimulates the level of cell cycle proteins. **(A–D)** Representative image of Western blotting as well as quantitative analysis of cyclin D1, CDK4, cyclin A2 and cyclin B1 denoted to be a fraction of GAPDH *in vitro* (*n* = 4). In addition, data are denoted to be mean ± SEM. ^#^ *p* < 0.05 in relative to the control group; *p < 0.05, **p < 0.01, ***p < 0.001 in relative to OGD group. **(E–H)** Representative image of Western blotting as well as quantitative analysis of cyclin D1, CDK4, cyclin A2 and cyclin B1 denoted to be a fraction of GAPDH *in vivo* (*n* = 4). Data are presented as the mean ± SEM. *p < 0.05, **p < 0.01, ***p < 0.001 VS AMI group.

Several cell cycle proteins are vital for generating cardiomyocytes (Ponnusamy et al., 2017). Cyclins and cyclin-dependent kinases (CDKs) are positive modulators of cell cycle progression. Their increased levels serve as an index of mitotic events in cardiomyocytes. To elucidate the mechanism underlying the effect of morroniside on induction of cardiomyocyte cell cycle activity, we evaluated the expression of cyclin D1, CDK4, cyclin A2, and cyclin B1 proteins using Western blotting. As shown in Figures 4A–D, OGD increased cyclin D1, CDK4, cyclin A2, and cyclin B1, and the differences between cyclin A2 and cyclin B1 were significant. Cyclin D1 expression was notably elevated in the 10 and 100 μM morroniside-treated groups relative to that in NRCMs in the OGD group (Figure 4A). There was also a tendency of increased CDK4 expression in the 100 μM morroniside-treated group relative to the OGD group (Figure 4B). Similar to the levels of cyclin D1 and CDK4, the expression of cyclin A2 was elevated in the 10 μM and 100 μM morroniside-treated groups relative to that in the AMI group (Figure 4C), and the level of cyclin B1 was increased in the 100 μM morroniside-treated group (Figure 4D). To validate the effects of morroniside on the expression of these cell cycle proteins, we assessed the expression of cyclin D1, CDK4, cyclin A2, and cyclin B1 in rats with AMI. As shown in Figures 4E–H, cyclin D1, CDK4, cyclin A2, and cyclin B1 levels were elevated in the AMI group, but not significantly. In comparison with that in the AMI group, the level of cyclin D1 in the 240 mg/kg morroniside-treated group increased obviously, and the 120 and 240 mg/kg morroniside-treated groups showed significant increases in CDK4, cyclin A2, and cyclin B1 levels relative to those in the AMI group. According to these results, proteins associated with the cell cycle mediate the effects of morroniside on cardiomyocytes following AMI.

### 3.4 Morroniside reduces myocardial fibrosis area and promotes heart repair

**FIGURE 5:**
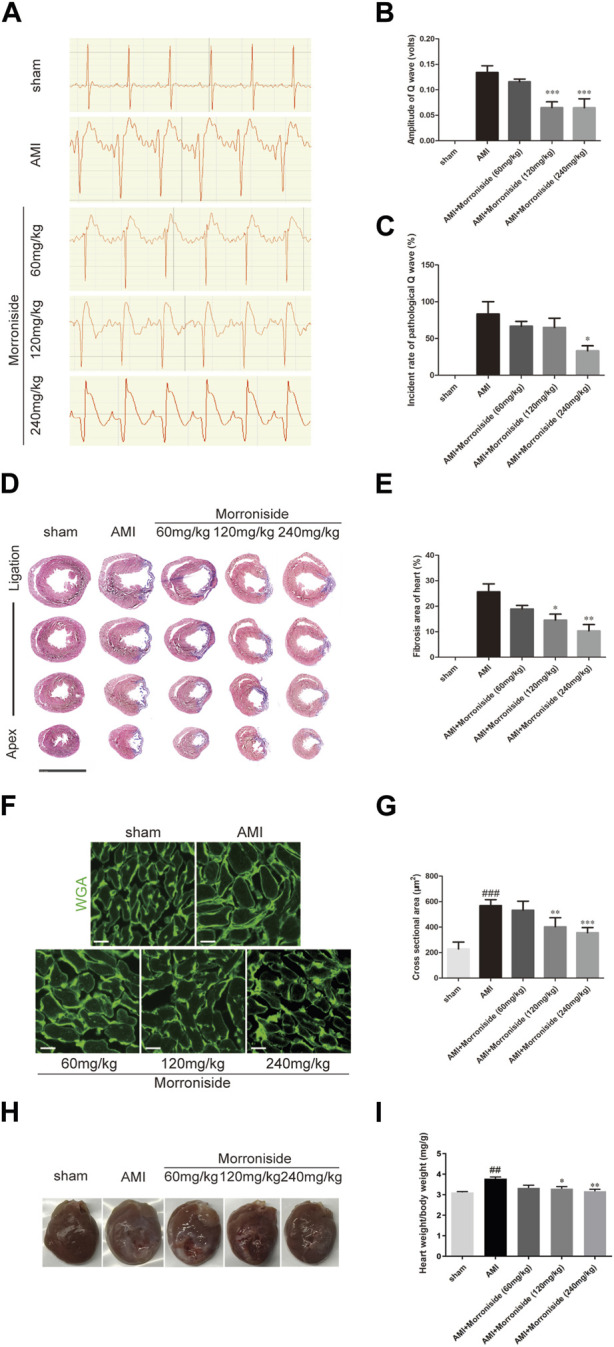
Morroniside reduces the size of myocardial infarction and improves heart tissue repair. **(A)** Representative image of ECG in rats at 7 days post-surgery. **(B)** Quantification of the amplitude of the Q wave (volts) (*n* = 4). **(C)** Quantification of the incidence of pathological Q wave (over 0.04 s of duration and with the amplitude of over 25% of the corresponding R wave; *n* = 3). **(D)** Masson’s trichrome-stained serial sections 28 days after AMI (*n* = 4). Scale bar = 10 mm. **(E)** Left ventricular scar size. Quantification of the fibrotic area compared with the myocardial area in trichrome-stained sections showed an obvious reduction in scar formation in the 120 mg/kg and 240 mg/kg morroniside-treated groups relative to the AMI group. **(F)** WGA staining showed a larger cardiomyocyte size in the AMI group than in sham group hearts at the border zone, and a smaller cardiomyocyte size in the 120 mg/kg and 240 mg/kg morroniside-treated groups than in the AMI group (*n* = 5). 3,000 cells were quantified per group. Scale bar = 20 μm. **(G)** The quantification was performed by exploring the transverse cell size **(H)** Representative images of hearts from each group. **(I)** The heart-to-body weight ratio was measured in each group. Data are presented as the mean ± SEM. ^###^ *p* < 0.001 relative to the sham group; when compared with AMI group, *p < 0.05, **p < 0.01, ***p < 0.001.

Finally, we evaluated the function of morroniside in cardiac repair using a rat model of AMI. As shown in Figure 5A, the sham group showed no abnormal ECG patterns. Compared with the sham group, a pathological Q wave was observed in the ECG of the AMI group 7 days post-surgery. Our data showed that the 120 and 240 mg/kg morroniside-treated groups presented an obvious reduction in Q wave amplitude relative to the AMI group (Figure 5B). Figure 5C illustrates the incidence rate of pathological Q waves 7 days post-surgery; the incidence in the 240 mg/kg morroniside-treated group was notably lower than that in the AMI group. Analysis of Masson’s trichrome-stained heart cross-sections and quantification of scar size at 28 days post-surgery demonstrated that the infarcts were notably smaller in the 120 and 240 mg/kg morroniside-treated groups than in the AMI group (Figures 5D, E). In addition, WGA staining revealed the size of the cardiomyocytes in the border zone. The findings demonstrated that the cardiomyocyte size was larger in the AMI group than in the sham group, and cardiomyocytes in the 120 mg/kg and 240 mg/kg morroniside-treated groups were smaller than those in the AMI group (Figures 5F, G), suggesting a reduction in cardiomyocyte hypertrophy. We also examined the ratio of heart to body weight. The results showed that morroniside treatment (120 or 240 mg/kg) resulted in a dramatic reduction in the heart-to-body weight ratio in rats with AMI compared to the model group (Figures 5H, I). These data demonstrate that morroniside improves myocardial fibrosis and hypertrophy, and promotes heart repair in rats with AMI.

## 4 Discussion

This study demonstrated the role of morroniside in induction of cardiomyocyte cell cycle activity and myocardial repair after injury. Several lines of evidence have demonstrated that morroniside induces the cell cycle activity of cardiomyocytes after OGD *in vitro* and after AMI *in vivo*. In addition, the mechanism of cardiomyocyte cell cycle activity underlying these effects may involve cell cycle proteins, including cyclin D1, CDK4, cyclin A2, and cyclin B1. Furthermore, morroniside improved myocardial fibrosis and cardiomyocyte hypertrophy 28 days after AMI.

AMI remains a significant cause of death worldwide; however, current treatments cannot replace lost cardiomyocytes (Virani et al., 2020). Recently, the field of myocardial regeneration has grown in popularity for the treatment of AMI (Polizzotti et al., 2015; Wang et al., 2017; Mohamed et al., 2018), and more studies have shown that myocardial regeneration is mainly driven by the proliferation of pre-existing cardiomyocytes (Porrello et al., 2011; Porrello et al., 2013; Mahmoud et al., 2014b). Our study revealed that morroniside induced cardiomyocyte cell cycle activity after AMI. Cardiac troponin T (cTnT) has been identified as a cytoplasmic marker (Hirose et al., 2019). Ki67 binds to nuclear antigens and is a robust marker of cell proliferation, which is expressed in all phases of the cell cycle except for G0 (Pathmanathan and Balleine, 2013). According to our findings, the percentage of newly generated cardiomyocytes labeled with cTnT and Ki67 was elevated after OGD. Morroniside treatment after OGD increased the number of Ki67^+^/cTnT^+^ NRCMs. We then added other markers of proliferating cells, including BrdU (S-phase) incorporation, pH3 (G2-phase/mitosis), and Aurora B (M-phase), to cTnT^+^ NRCMs after OGD. The results revealed that morroniside increased the number of BrdU^+^/cTnT^+^, pH3^+^/cTnT^+^, and Aurora B^+^/cTnT^+^ NRCMs, which was consistent with the increasing tendency of Ki67^+^/cTnT^+^ cells. We primarily showed that morroniside stimulated the cell cycle activity of NRCMs after OGD. Using a rat model of acute myocardial infarction, we investigated the effects of morroniside on cell cycle activity *in vivo*. In support of the *in vitro* findings, the *in vivo* findings demonstrated that morroniside-treated groups had elevated Ki67^+^/cTnT^+^, BrdU^+^/cTnT^+^, pH3^+^/cTnT^+^, and Aurora B^+^/α-actinin^+^ cells in the peri-infarct areas after AMI.

This study further verified whether the effect of morroniside on cell cycle activity improved heart repair after AMI. ECG has a vital impact on the diagnosis of AMI, owing to its universal availability and low cost. Following the acute period, the ECG pattern may present pathological Q waves (over 0.04 s duration and with an amplitude of over 25% of the corresponding R wave; Delewi et al., 2013). The appearance of Q waves on an ECG is of great value for the clinical prognosis. These findings demonstrate that morroniside reduced the amplitude and incidence rate of pathological Q waves, suggesting a reduction in the area of the myocardium that could not be depolarized. Masson’s trichrome staining demonstrated that morroniside reduced the area of myocardial fibrosis, which may be associated with its effects on cell cycle activity. The injury-induced remodeling of adult myocardial tissue results from reduced cardiomyocyte replenishment (Porrello et al., 2011). WGA staining revealed that morroniside reduced myocardial hypertrophy, an important pathological factor that leads to cardiac insufficiency and arrhythmia. In our previous study on angiogenesis, echocardiography was performed on the rats to evaluate alterations in cardiac structure and function. The result showed that treatment with morroniside significantly augmented the LVEF and LVFS 7 days, 14 days and 28 days post-surgery (Liu et al., 2020). Our study did not fully demonstrate that the improvement in cardiac function was due to cell cycle activity. Therefore, it is essential to perform further studies to explore the mechanism of action of this drug in detail. In addition, its biologically active components must be explored.

Several sources of mitogenic signals that induce cell cycle activity have been identified in the heart, including the Hippo signaling pathway, the Neuregulin (NRG1)-ErbB pathway, the Notch signaling pathway and the Wnt/β-catenin signaling pathway, etc (Yin et al., 2021). These signaling pathways often play a role in promoting myocardial cell proliferation after myocardial infarction by regulating the cell cycle, which is considered as a “molecular switch”. Genetic triggers for cell cycle reactivation to promote cardiomyocyte mitosis in the adult heart have been advanced as potential therapeutic targets for cardiac regeneration (Broughton et al., 2018). Therefore, we preliminarily observed the effect of morroniside on the expression levels of cell cycle proteins. Cyclins and CDKs are positive regulators of cell cycle progression, and their increase is an indicator of mitotic events in all cell types (Ponnusamy et al., 2017). Molecular studies have shown that cyclin D1 and CDK4 play vital roles in cell cycle activity (Mohamed et al., 2018; Hashmi and Ahmad, 2019; Abouleisa et al., 2022). To explain the mechanism underlying the effect of morroniside on cardiomyocytes, the present study assessed the levels of proteins related to the cyclin D1/CDK4 signaling pathway using Western blotting. Our findings revealed that morroniside treatment elevated the expression of these proteins above that in the OGD group. In addition, G2/M-phase cyclins (cyclins A and B) can be found in embryonic and neonatal cardiomyocytes, but are undetectable in young and adult hearts (Porrello et al., 2011; Puente et al., 2014). Studies have shown that cyclin A2 (Yester and kühn, 2017) and cyclin B1 (Mohamed et al., 2018; Hashmi and Ahmad, 2019) drive G1/S and G2/M phases to promote mammalian cardiomyocyte cell cycle activity. We also amined the protein levels of cyclin A2 and B1. The expression of cyclins A2 and B1 was higher in the morroniside-treated groups than in the OGD group, which was similar to the levels of cyclin D1 and CDK4. Furthermore, we determined the regulation of these cell cycle proteins by morroniside at the animal level, using an *in vivo* AMI model. The *in vitro* results demonstrated that the morroniside treatment groups had elevated levels of cyclin D1, CDK4, cyclin A2, and cyclin B1 compared to the AMI group. Thus, the mechanism by which morroniside stimulates cell cycle activity may be mediated by cell cycle proteins. However, further investigation is still required to explore the specific molecular targets of morroniside and elucidate the underlying molecular mechanisms involved.

## 5 Conclusion

Collectively, we demonstrated that morroniside induced cardiomyocyte cell cycle activity and promoted cardiac repair after AMI. The morroniside-induced enhanced expression of proteins associated with cell cycle progression mediated cardiomyocyte cell cycle activity.

## Data Availability

The original contributions presented in the study are included in the article/Supplementary Material, further inquiries can be directed to the corresponding author.

## Abbreviations

AMI, Acute myocardial infarction; BrdU, 5-bromo-2-deoxyuridine; CDKs, Cyclin-dependent kinases; CDK4, Cyclin-dependent kinase 4; DAPI, diamidino-2-phenylinidole; DMEM, Dulbecco’s modified Eagle’s medium; ECG, Electrocardiography; FBS, Fetal bovine serum; HPLC, High-performance liquid chromatography; LAD, Ligated the left anterior descending; NRCMs, Neonatal rat cardiomyocytes; OCT, Optimal cutting temperature; OGD, Oxygen-glucose deprivation; PBS, Phosphate-buffered saline; PFA, Paraformaldehyde; pH3, phospho-histone H3; SEM, Standard Error of Mean; WGA, Wheat germ agglutinin.
